# Supplementary material for: Cortical excitability controls the strength of mental imagery
Source: eLife. 2020 May 5;9:e50232. doi: 10.7554/eLife.50232 (PMC7200162; doi:10.7554/eLife.50232)
Supplement: Source code 1. [file elife-50232-code1.rtf]

R code for mixed linear models - Written by Rebecca Keogh - lme4 packagelibrary (“lme4”)setwd ("~/Desktop/Manuscripts/tDCS_Imagery/tdcs_imagery”) % Sets path - use cd of where csv is%% Can also do this by going to misc and changing by handdata = read.csv("data.csv") % Loads data which(!complete.cases(data)) % Shows which have missing data%%make variable categoricaldata$order = factor(c(data$order))data$block = factor(c(data$block))data$tDCS = factor(c(data$tDCS))%%checkdata$order data$blockdata$tDCS %% Only intercept not slopetdcs.model = lmer(imagery ~ tDCS + block + order + (1|subject) ,data=data, REML=FALSE) %Runs LME on data - subjects is random effect with intercept + slope controlled for - rest are fixedsummary(tdcs.model) %Prints model variables etctdcs.null = lmer(imagery ~ block + order + (1|subject),data=data, REML=FALSE) %Runs LME on null model - subjects is random effect with intercept + slope controlled for - rest are fixedsummary(tdcs.null) %Prints model variables etcAnova%% Assumptionsplot(fitted(tdcs.model),residuals(tdcs.model)) % Linearity want to see random spatter of blobs dots - also homoscadaictyhist(residuals(tdcs.model)) - normality qqnorm(residuals(tdcs.model)) - normality - should form diagonal lineanova(tdcs.model,tdcs.null)
