# Supplementary material for: Cortical excitability controls the strength of mental imagery
Source: eLife. 2020 May 5;9:e50232. doi: 10.7554/eLife.50232 (PMC7200162; doi:10.7554/eLife.50232)
Supplement: Supplementary file 1. [file elife-50232-supp1.docx]

**SUPPLEMENTARY MATERIAL**

**Cortical excitability controls the strength of mental imagery**

- **Supplementary Tables (5 Tables)**

**Supplementary Tables**

**Supplementary Table S1**

Surface-based whole brain analysis of the fMRI resting-state data (TR=2s): Corrected clusters showing a significantly *negative* association with individual imagery strength at a cluster-wise probability threshold of *P* < .05 (also see **Supplementary Fig. S1**).

| Cluster No. | Maximum voxel-wise significance in cluster | Peak vertex annotation | Size (mm^2^) | MNI 305 coordinates of peak vertex | | | Cluster-wise *P*-value | NVtxs | Laterality | Area label |
| --- | --- | --- | --- | --- | --- | --- | --- | --- | --- | --- |
| Main fMRI experiment, TR = 2 seconds | | | | | | | | | | |
| 1 | -4.613 | 11477 | 552.22 | -24.7 | -86.6 | 12.8 | 0.0002 | 903 | L | lateraloccipital |
| 2 | -4.172 | 97048 | 535.7 | -20.6 | -96.9 | 3.3 | 0.0002 | 734 | L | lateraloccipital |
| 3 | -3.936 | 130756 | 534.22 | -13.3 | -86.4 | 5.3 | 0.0002 | 698 | L | pericalcarine |
| 4 | -3.053 | 63050 | 248.57 | -19.5 | -80.4 | -7.3 | 0.0024 | 298 | L | lingual |
| 5 | -2.848 | 162967 | 203.8 | -31.3 | -59.6 | -8.4 | 0.01276 | 374 | L | fusiform |
| 1 | -4.488 | 69964 | 301.97 | 17 | -96.6 | 0.8 | 0.0004 | 408 | R | pericalcarine |
| 2 | -4.021 | 71800 | 560.64 | 27.4 | -92.3 | 1.2 | 0.0002 | 785 | R | lateraloccipital |
| 3 | -3.739 | 117794 | 239.69 | 14.6 | -91.3 | 19 | 0.0028 | 301 | R | lateraloccipital |
| 4 | -2.502 | 104614 | 214.22 | 35.9 | -82.5 | 18.4 | 0.00519 | 366 | R | inferiorparietal |
| Additional fMRI experiment, TR = 1 second | | | | | | | | | | |
| 1 | -3.934 | 11477 | 214.78 | -24.7 | -86.6 | 12.8 | 0.00878 | 361 | L | lateraloccipital |
| 2 | -2.191 | 27912 | 192.84 | -7.6 | -98.8 | 6.2 | 0.01911 | 230 | L | lateraloccipital |
| 3 | -2.167 | 46458 | 228.65 | -24.2 | -73.6 | -4.6 | 0.00599 | 387 | L | lingual |
| 1 | -4.128 | 71801 | 378.06 | 27.5 | -93 | 0.9 | 0.0002 | 543 | R | lateraloccipital |
| 2 | -4.106 | 101052 | 430.75 | 5.3 | -80.8 | 13.6 | 0.0002 | 574 | R | cuneus |
| 3 | -3.07 | 117793 | 195.84 | 14.4 | -90.7 | 19.4 | 0.01157 | 245 | R | lateraloccipital |
| 4 | -2.798 | 90715 | 216.85 | 24 | -78.5 | -7 | 0.00499 | 364 | R | lateraloccipital |

Note: Correction for multiple comparisons at a cluster-wise probability threshold of *p* < .05 was done using a pre-cached Monte Carlo Null-Z simulation with 10000 iterations. The locations of the clusters strongly overlap with the locations of the atlas- and retinotopically defined areas that showed a negative association with imagery strength. Abbreviations: L = left hemisphere, R = right hemisphere; NVtxs = Number of vertices.

**Supplementary Table S2**

Surface-based whole brain analysis of the fMRI resting-state data (TR=2s): Corrected clusters showing a significantly *positive* association with individual imagery strength at a cluster-wise probability threshold of *P* < .05 (also see **Supplementary Fig. S2**).

| Cluster No. | Maximum voxel-wise significance in cluster | Peak vertex annotation | Size (mm^2^) | MNI 305 coordinates of peak vertex | | | Cluster-wise *P*-value | NVtxs | Laterality | Area label (of peak vertex) |
| --- | --- | --- | --- | --- | --- | --- | --- | --- | --- | --- |
| Main fMRI experiment, TR = 2 seconds | | | | | | | | | | |
| 1 | 3.775 | 108491 | 470.82 | -52.7 | -21.2 | 33.4 | 0.0002 | 1019 | L | postcentral |
| 2 | 3.749 | 95009 | 440.42 | -40.1 | -13.9 | 20.6 | 0.0002 | 1163 | L | insula |
| 3 | 3.715 | 121748 | 1668.23 | -43.2 | 29.7 | 25.7 | 0.0002 | 2992 | L | rostralmiddlefrontal |
| 4 | 3.463 | 82836 | 192.99 | -8.3 | 31.5 | 53.6 | 0.01514 | 368 | L | superiorfrontal |
| 5 | 3.26 | 82794 | 292.83 | -17.5 | 27.1 | 50.2 | 0.0004 | 462 | L | superiorfrontal |
| 6 | 3.171 | 119359 | 377.67 | -47.1 | -9.1 | 24.9 | 0.0002 | 928 | L | postcentral |
| 7 | 2.908 | 129777 | 250.18 | -7.3 | 53.6 | -6.2 | 0.0016 | 315 | L | medialorbitofrontal |
| 8 | 2.897 | 34901 | 238.38 | -7.1 | 56 | 25.3 | 0.0026 | 382 | L | superiorfrontal |
| 9 | 2.848 | 117187 | 597.68 | -9.9 | 30 | 31.1 | 0.0002 | 1199 | L | superiorfrontal |
| 10 | 2.180 | 102903 | 277.18 | -35.3 | 13.6 | -4.9 | 0.0004 | 747 | L | insula |
| 11 | 1.886 | 153160 | 175.60 | -11.6 | 49.8 | 7.8 | 0.03096 | 298 | L | superiorfrontal |
| 1 | 4.499 | 102470 | 1518.23 | 44.3 | 31.2 | 21.4 | 0.0002 | 2730 | R | rostralmiddlefrontal |
| 2 | 3.291 | 48386 | 195.37 | 23 | 0.5 | 60 | 0.01236 | 435 | R | superiorfrontal |
| 3 | 2.698 | 51226 | 417.29 | 8.3 | 61.7 | -0.8 | 0.0002 | 572 | R | superiorfrontal |
| 4 | 2.445 | 46598 | 185.98 | 17.4 | -10.8 | 60.3 | 0.01851 | 377 | R | precentral |
| Additional fMRI experiment, TR = 1 second | | | | | | | | | | |
| 1 | 3.619 | 71944 | 925.62 | -52.7 | -0.1 | 34.1 | 0.0002 | 1747 | L | precentral |
| 2 | 3.482 | 121086 | 328.11 | -51 | -8 | 43.8 | 0.0002 | 743 | L | precentral |
| 3 | 3.192 | 57217 | 335.35 | -59.6 | -31 | 36 | 0.0002 | 777 | L | supramarginal |
| 4 | 3.141 | 75151 | 193.7 | -59.5 | -7.3 | -3 | 0.01415 | 375 | L | superiortemporal |
| 5 | 3.035 | 73600 | 202.86 | -35.9 | -6.1 | 16.8 | 0.00997 | 621 | L | insula |
| 6 | 1.832 | 51450 | 177.35 | -35.1 | -18.9 | -2.4 | 0.02899 | 390 | L | insula |
| 1 | 4.683 | 50531 | 1912.42 | 43.8 | 30.2 | 30.3 | 0.0002 | 3369 | R | rostralmiddlefrontal |
| 2 | 3.2 | 124217 | 191.25 | 40.8 | 12.8 | 47.7 | 0.01475 | 268 | R | caudalmiddlefrontal |
| 3 | 3.156 | 8870 | 287.03 | 46.9 | -14.4 | 30.9 | 0.0002 | 731 | R | Postcentral |
| 4 | 2.598 | 60827 | 318.92 | 9.8 | 58.8 | 0.1 | 0.0002 | 411 | R | superiorfrontal |

Note: Correction for multiple comparisons at a cluster-wise probability threshold of *p* < .05 was done using a pre-cached Monte Carlo Null-Z simulation with 10000 iterations. The locations of the clusters are strongly overlapping with the locations of the atlas-defined areas that showed a positive association with imagery strength. Abbreviations: L = left hemisphere, R = right hemisphere; NVtxs = Number of vertices.

**Supplementary Table S3**

Surface-based whole brain analysis of the fMRI resting-state data (TR=2s): Corrected clusters showing significantly positive and negative associations with individual subjective vividness at a cluster-wise probability threshold of *P* < .05 (also see **Supplementary Fig. S3**).

| Cluster No. | Maximum voxel-wise significance in cluster | Peak vertex annotation | Size (mm^2^) | MNI 305 coordinates of peak vertex | | | Cluster-wise *P*-value | NVtxs | Laterality | Area label (of peak vertex) |
| --- | --- | --- | --- | --- | --- | --- | --- | --- | --- | --- |
| 1 | -3.067 | 21503 | 185.66 | -10.9 | -78.5 | 24.4 | 0.02405 | 239 | L | cuneus |
| 2 | 2.526 | 42871 | 290.45 | -31.3 | 32 | 33.7 | 0.0004 | 480 | L | rostralmiddlefrontal |

Note: Correction for multiple comparisons at a cluster-wise probability threshold of *p* < .05 was done using a pre-cached Monte Carlo Null-Z simulation with 10000 iterations. Abbreviations: L = left hemisphere, R = right hemisphere; NVtxs = Number of vertices.

**Supplementary Table 4**

Correlations between normalized fMRI mean intensity data (TR=2 seconds) of visual cortex ROIs and whole-brain anatomical measures as a proxy for head size. The anatomical measures are taken from the data published in [1] . Early visual cortices V1, V2 and V3 were functionally defined, while lateral occipital cortex was atlas-defined. Values denote Pearson product moment correlations; values in brackets denote the respective p-value. ** = p-value < .01, * = p-value < .05.

|  | Total cortical surface size | Total cortical thickness | Total Ccrtical volume |
| --- | --- | --- | --- |
| V1 normalized mean fMRI intensity | -.023 (.903) | .144 (439) | .055 (.769) |
| V2 normalized mean fMRI intensity | -.258 (.162) | .082(.662) | -.184 (.323) |
| V3 normalized mean fMRI intensity | -.122(.514) | .281 (.125) | .010 (.957) |
| Lateral occipital cortex normalized mean fMRI intensity | .168 (.365) | .306 (.094) | .249 (.176) |

**Supplementary Table 5**

Correlations between normalized fMRI mean intensity data (TR=2 seconds) of visual cortex ROIs and their respective anatomical measures. The anatomical measures are taken from the data published in [1]. Early visual cortices V1, V2 and V3 were functionally defined; the respective anatomical measures refer to the central area (i.e. the regions representing the central visual field). Lateral occipital cortex was atlas-defined. Values denote Pearson product moment correlations; values in brackets denote the respective p-value. ** = p-value < .01, * = p-value < .05.

|  | Surface size of respective area | Cortical thickness of respective area | Cortical volume of respective area |
| --- | --- | --- | --- |
| V1 normalized mean fMRI intensity | .503 (.004) ** | -.408 (.023)* | .339 (.062) |
| V2 normalized mean fMRI intensity | .346 (.057) | .163 (.381) | .369 (.041)* |
| V3 normalized mean fMRI intensity | -.003 (.986) | .230 (.213) | .124 (.506) |
| Lateral occipital cortex normalized mean fMRI intensity | .231 (.211) | .337 (.064) | .341 (.060) |

**References**

1. Bergmann, J., et al., *Smaller Primary Visual Cortex Is Associated with Stronger, but Less Precise Mental Imagery.* Cereb Cortex, 2016. **26**(9): p. 3838-50.
